# Analyzing the Effects of Gap Junction Blockade on Neural Synchrony via a Motoneuron Network Computational Model

**DOI:** 10.1155/2012/575129

**Published:** 2012-12-04

**Authors:** Heraldo Memelli, Kyle G. Horn, Larry D. Wittie, Irene C. Solomon

**Affiliations:** ^1^Department of Computer Science, Stony Brook University, Stony Brook, NY 11794-4440, USA; ^2^Department of Physiology & Biophysics, Stony Brook University, Stony Brook, NY 11794-8661, USA; ^3^Program in Neuroscience, SUNY, Stony Brook University, Stony Brook, NY 11794-5230, USA

## Abstract

In specific regions of the central nervous system (CNS), gap junctions have been shown to participate in neuronal synchrony. Amongst the CNS regions identified, some populations of brainstem motoneurons are known to be coupled by gap junctions. The application of various gap junction blockers to these motoneuron populations, however, has led to mixed results regarding their synchronous firing behavior, with some studies reporting a decrease in synchrony while others surprisingly find an increase in synchrony. To address this discrepancy, we employ a neuronal network model of Hodgkin-Huxley-style motoneurons connected by gap junctions. Using this model, we implement a series of simulations and rigorously analyze their outcome, including the calculation of a measure of neuronal synchrony. Our simulations demonstrate that under specific conditions, uncoupling of gap junctions is capable of producing either a decrease or an increase in neuronal synchrony. Subsequently, these simulations provide mechanistic insight into these different outcomes.

## 1. Introduction

Gap junctions are found in a number of areas in the mammalian CNS and are believed to play a significant role in neuronal synchrony [1, 2]. Gap junctions link the intracellular space of two neurons, permitting ions and metabolic molecules to pass between neighboring cells, resulting in a coupling of both electrical and metabolic behavior [3, 4]. These junctions are formed from a hexameric assembly of structural proteins called connexins (Cx), and a number of Cx isoforms, including Cx26, Cx32, Cx36, Cx30.2, Cx45, and Cx50, have been identified in some populations of neurons [5–15]. Of these Cx isoforms, Cx26, Cx32, and Cx36 have been reported to be expressed in neurons and/or motoneurons in respiratory-related CNS regions [9, 11, 12, 14, 16–20]. 

While many CNS regions have been shown to express Cx proteins or functional gap junction coupling, gap junctions are often present in areas where synchronized firing activity is important. Amongst these CNS regions, brainstem areas associated with central respiratory control (including respiratory-related hypoglossal and phrenic motoneurons), have been shown to express Cx proteins [12, 14, 16–18, 20] or functional coupling [21–23]. Moreover, blockade of gap junctions has been shown to alter not only respiratory activity but also inspiratory-phase neuronal synchrony [24, 25], an observation that is consistent with the idea that the conductance and opening or closing of gap junctions has a direct effect on synchrony of neuronal networks [26]. 

Intuitively, one might assume that gap junction blockers would produce a complementary decrease in neural synchrony; however, studies examining the effects of gap junction blockade have produced mixed results. In the field of central respiratory control, this is highlighted by a series of studies focusing on respiratory rhythm generation and inspiratory-phase neuronal synchrony. In these studies, Solomon et al. [25] demonstrated that pharmacological blockade of brainstem gap junctions reduces inspiratory-phase synchronization in the phrenic nerve in the adult rat while Bou-Flores and Berger [24] showed that on a short-time-scale, gap junction blockade increased inspiratory-phase synchronization in the hypoglossal and phrenic nerves in the neonatal rat. Additionally, Winmill and Hedrick [27] reported that fictive breathing was differentially affected by blockade of gap junctions in larval versus adult bullfrogs. While age-related differences in Cx expression and gap junction coupling are known to exist [4, 7, 9, 12, 16, 17, 20, 28] it is unclear how or why neuronal synchrony would be differentially affected by blockade of gap junctions in the above studies.

To address these curious and conflicting findings in the literature, we have opted to take a computational approach, in the hopes of elucidating potential mechanisms that might explain the gap junction-mediated decreases versus increases in neuronal synchrony. Using a Hodgkin-Huxley style neuronal network model of motoneurons, connected to each other via gap junctions, we make changes to gap junction conductance to emulate the experimental application of pharmacological gap junction blockers. In addition, we performed a wide range of computer simulations and analyzed various parameters to understand the effects of gap junction blockade on synchrony.

Ultimately, we observed that it is possible to obtain either a decrease or an increase in synchronized firing activity, or even eliminate excitability altogether, based entirely on modifications to gap junction conductance and excitatory inputs into our model. The motivation for altering excitatory input is based on the possibility that the gap junction blockers affect areas outside of the nucleus under study, which we deem to be of critical importance when discussing changes in synchrony.

## 2. Methods and Simulation Details

**Figure 1:**
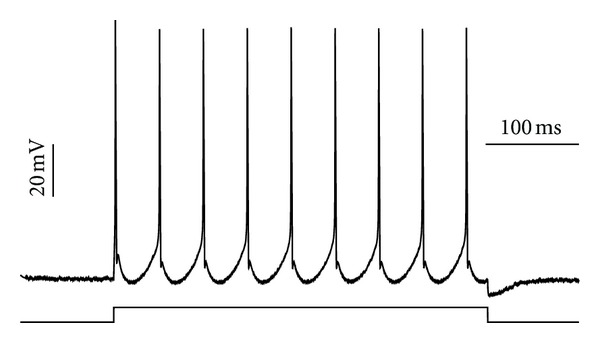
Example of our HM single-neuron model firing under a square stimulus current of *I*_input_ = 0.6 nA.

The model was coded entirely in C++ and all simulations were run on a 2011 Macbook Pro laptop. Graphs were created with Python's MatPlotLib plotting library [29]. The neuron model used in all simulations was a numerically integrated Hodgkin-Huxley-style model based on a series of differential equations for the hypoglossal motoneuron (HM) generated by Purvis and Butera [30]. Unless otherwise stated, all of the model parameters are identical to the original Purvis and Butera model [30]. It is a single-compartment (isopotential) electrophysiological model based on experimental data from neonatal rats that reproduces detailed features of its biological counterpart. Figure 1 provides an example of action potential firing from the implemented single-cell model. Despite using a very specific motoneuron for our simulations, we believe that the network-level behaviors discussed in this paper apply to other similar neuronal networks, and the HM shares a number of common features with many other neuron models [23].

For our model, the HMs were probabilistically connected into a network via simple bidirectional nonvoltage-dependent gap junctions, and their implementation was based on the model by Perez Velazquez and Carlen [28]. The vast majority of the simulations that were run consisted of a network of 100 neurons, with a connection probability of 20% (i.e., each neuron is coupled by gap junctions with 20 other neurons on average). If neuron *i* is connected to neuron *j* it receives a current of −*g*(*V*_*i*_ − *V*_*j*_) from neuron *j*, where *g* is the gap junction conductance. Thus, a higher degree of connectivity would lead to a greater total current into the neuron (as a result of the greater number of gap junctions with other neurons) and a higher degree of coupling, but this is beyond the scope of this paper.

For our simulations, Euler's method was used for numerical integration with a constant time step of 0.05 ms; few of the simulations were checked at a *dt* = 0.01 ms to ensure correctness and numerical stability. A square-wave excitatory input current was applied to each neuron with average amplitude of 0.7 nA, and variable white noise was added to each neuron at every time step.

Gap junction conductance (*g*) was set to 2 nS in the “open” state, a value comparable to biological measurements of conductance. In order to simulate pharmacological blockade of gap junctions, we gradually lowered the gap junction conductance during the simulations. In addition, we ran separate simulations where the only difference in parameters was a decrease of the gap junction conductance. The simulated biological time was in the range of 5–30 seconds, and select portions of these simulations are shown. 

To evaluate synchrony, we implemented a quantitative measurement of network synchrony that we call *χ*, originally proposed by Hansel et al. [31]. This synchrony measure is computed by calculating the ratio of the time-averaged variance of the population voltage and the population average of time-averaged variance of single cell voltage. However, unlike the standard form, which is applied to data points over all time, we apply the measure to short time bins of 200 ms duration consecutively. Much like a short-time Fourier transform, this provides insight into how the properties of the signal change over time and helps to ameliorate limitations of a method that was originally intended for use over the entire signal:

(1) $$\begin{array}{c} \chi =\frac{\bar{{\left(\left(1/N\right)\sum_{i=1}^{N}V_{i}\left(t\right)\right)}^{2}}-{\bar{\left(\left(1/N\right)\sum_{i=1}^{N}V_{i}\left(t\right)\right)}}^{2}}{\left(1/N\right)\sum_{i=1}^{N}\left(\bar{V_{i}{\left(t\right)}^{2}}-{\left(\bar{V_{i}\left(t\right)}\right)}^{2}\right)}. \end{array}$$

We also determined the average firing frequency to further characterize the properties of the entire nucleus. For this measure, we first take a time window of 1000 ms. Then, we determine the average firing frequency for each neuron separately within that time window. Finally, we calculate the mean and standard deviation for these averages, which yield statistical measures of firing frequency over the entire simulated nucleus. 

## 3. Results

### 3.1. Basic Properties

**Figure 2:**
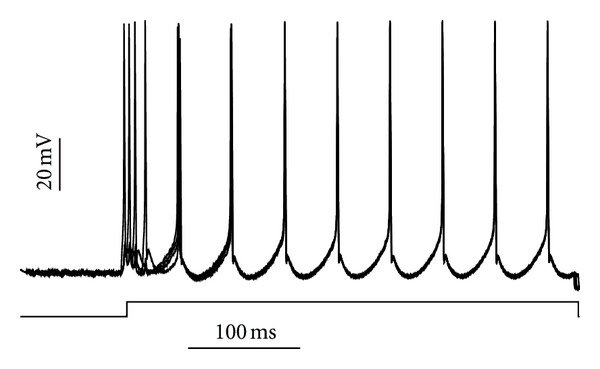
Example of 4 HMs connected with each other via gap junctions. The neurons rapidly attain perfect synchrony following opening of the gap junctions.

We started our simulations with a demonstration of the basic properties of our model. To illustrate the behavior of connecting motoneurons via gap junctions, we provide an example of a simple network of 4 motoneurons coupled by high-conductance (4 nS) gap junctions (Figure 2). We start the simulation with “closed” gap junctions (conductance = 0) and then we open them to allow the flow of current between the cells. This simulation demonstrates that upon opening the gap junctions, the model rapidly comes to perfect synchrony, as expected. In this example, the stimulation current was applied prior to opening the gap junctions in order to demonstrate nonsynchronous firing prior to gap junction coupling.

### 3.2. Gap Junction Blockade of Motoneuron Nucleus Alone

**Figure 3:**
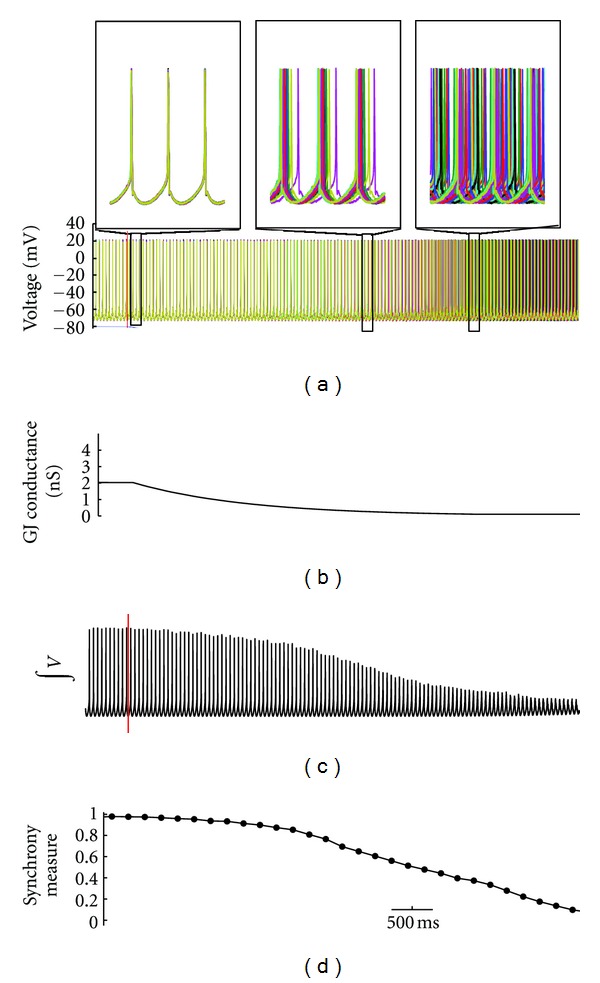
Example of simulation showing the effects of gap junction uncoupling. The biological simulated time was 8 seconds on a network of 100 motoneurons. (a) Voltage traces from a selection of 20 of the neurons; upper panel shows expanded traces from 3 regions indicated, demonstrating perfect synchrony during the initial segment, the reduction of synchrony as the neurons are uncoupling during the second segment, and unsynchronized firing in the final segment. (b) Average gap junction conductance (in nS). (c) Integrated total voltage trace of the entire network. (d) The measure of synchrony of the network with data points every 200 ms.

To simulate the gap junction blockade experiments, we first establish a gap junction coupled 100 motoneuron model. After simulating this model until full synchrony is achieved, gap junction conductance was gradually lowered, but never reduced to zero, since some of the pharmacological agents used to block gap junction coupling may only partially reduce channel conductance albeit other pharmacological agents completely close the channel (reviewed by Rozental et al. 2001 [32]). An example from this simulation is shown in Figure 3. As gap junction conductance decreases, so does neuronal synchrony. The decrease in synchrony is clearly seen when all voltage traces are summed together (Figure 3(c)), although the decrease in total voltage may not necessarily reflect a loss of synchrony.

To verify that a decrease in synchrony and not firing frequency was responsible for the changes observed in total voltage, we computed the neural synchrony measure described (Figure 3(d)) and determined the average firing frequency of neurons* before* gap junction conductance was decreased, *during* the decrease, and *late* in the decrease (see Figure 3(a) and Table 1). These procedures revealed that during gap junction blockade, there was a decrease in both the total voltage and the synchrony measure, but not in firing frequency. Thus, the decreases in total voltage and synchrony did not appear to be a byproduct of changes in firing frequency, as firing frequency remained constant over the course of the simulation (Table 1). These findings suggest that changes in synchrony are due to the alignment of spikes alone. 

### 3.3. Gap Junction Blockade of Motoneuron Nucleus and Upstream Inputs

**Figure 4:**
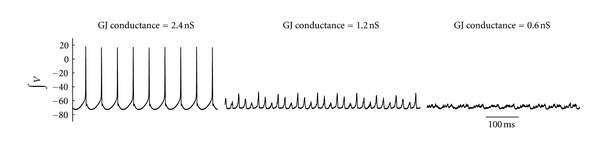
Lowering the gap junction conductance decreases the integrated voltage output.

Under experimental conditions in which the application of gap junction blockers is provided by bath application or systemic perfusion, the effects of gap junction blockade may not be exclusive to the neuronal population under investigation but could be influenced by other neural areas that provide direct or indirect input to this region. In the respiratory circuit, for example, some respiratory-related neurons in addition to the HMs have been shown to exhibit gap junction coupling. This includes the pre-BötC [23], which is the primary locus of inspiratory activity and as such is the major component of inspiratory drive to which hypoglossal motor activity is entrained. Thus, a decrease in gap junction coupling in the pre-BötC, which is upstream of the HMs, could alter HM activity since blocking gap junctions would be expected to decrease the total voltage (Figure 4) from this region as well. If this were to occur, it would lead to an alteration in the strength of the input to the HMs. To assess this possibility, upstream gap junction blockade was incorporated into the model as a reduction of the input current to the motor nucleus. 

**Figure 5:**
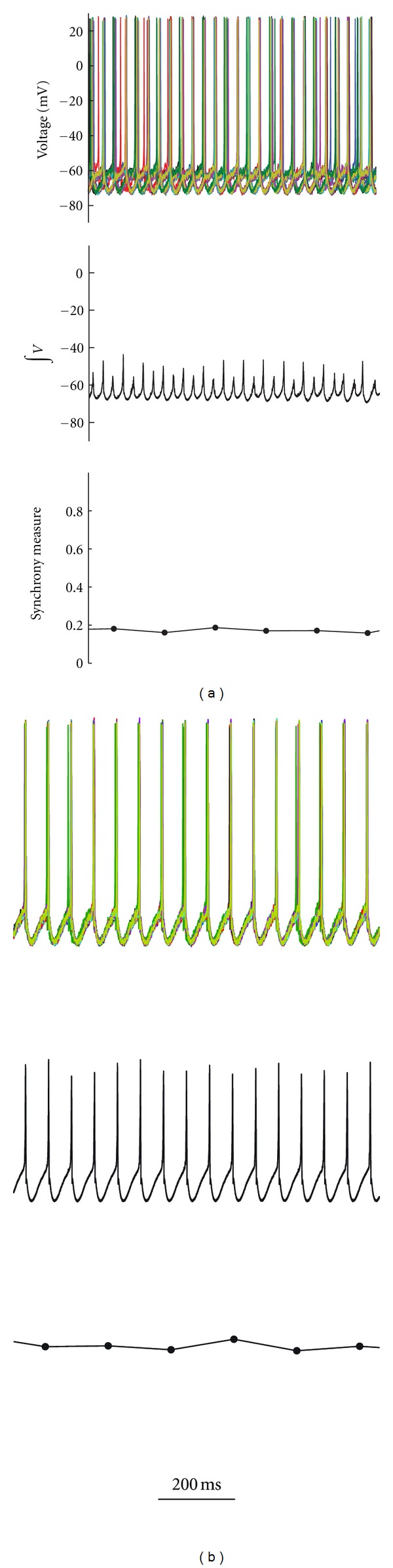
Reducing the input current (*I*) and gap junction conductance enhances neuronal synchrony. In (a) *I* = 0.5 nA and gap-junction conductance = 1.2 nS; in (b) *I* = 0.3 nA and gap-junction conductance = 0.8 nS.

 Thus, for these simulations, both the motoneuron nucleus and its upstream drivers were subjected to gap junction blockade by simultaneously reducing input current and gap junction conductance. Under these conditions, we observed effects that were distinct from those shown during gap junction blockade of the motoneuron nucleus alone (Section 3.2). In this case, rather than a decrease in synchrony, an increase in synchrony is observed as gap junction conductance is steadily decreased (Figure 5(a)). Concomitantly, the measure of neural synchrony is also increased, verifying that synchrony increases as gap junction conductance is reduced (Figure 5(b)).

As with the previous simulation, firing frequency statistics were determined to ensure that the observed change in synchrony was not an effect of changes in firing frequency. These statistics are summarized in Table 2.

**Figure 6:**
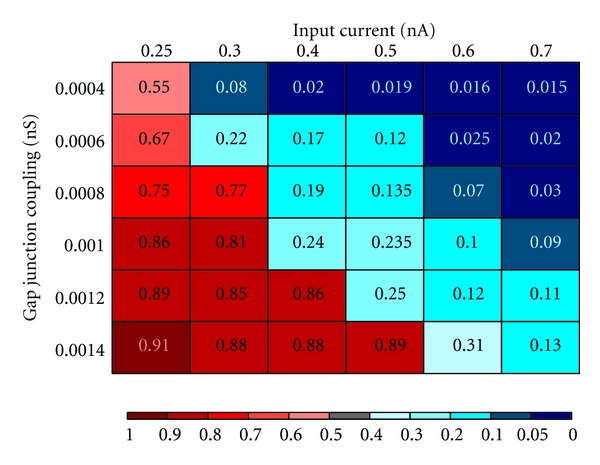
Effect of changes to gap-junction conductance and input current on neuronal synchrony.

To ensure that this effect is not an exotic behavior contingent on precise parameter settings, we ran several simulations over a broad variety of input currents and gap junction conductances. These simulations are summarized in Figure 6 and clearly demonstrate that synchrony increases monotonically with increases in gap junction conductance and fixed input current and decreases monotonically with increases in input current and fixed gap junction conductance.

Changing both gap junction conductance and input current could therefore either produce an increase or decrease in synchrony, depending on the initial synchrony state of the system and whether the change to gap junction conductance or input current was greater.

### 3.4. Gap Junction Conductance, Firing Threshold, and Excitability

**Figure 7:**
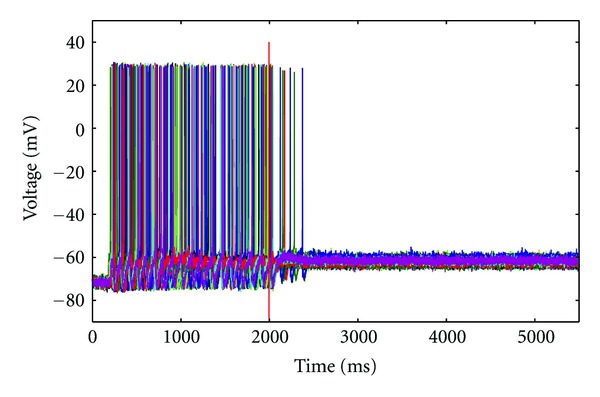
Example from the 100 motoneuron network showing the effects of opening gap junctions when input current is low. In this simulation, the input current was set at *I* = 0.2 nA, which is approximately rheobase for a single HM, and the gap junctions were initially almost fully closed. At *t* = 2000 ms, gap junctions were opened, which rapidly resulted in the cessation of firing.

Increasing and decreasing synchrony are not the only behaviors that can result from modifying gap junction conductance and input current. While the above simulations have focused on the effects of gap junction blockade, we also examined the influence of increasing gap junction conductance (i.e., opening gap junctions) on neuronal synchrony. For this simulation, however, our 100-motoneuron model was given an input current that is close to rheobase for a single neuron. In this case, increasing gap junction conductance was capable of eliminating firing altogether (Figure 7). While this observation may appear to be in contrast with our findings demonstrating that firing frequency typically remains stable when changing gap junction conductance, it highlights the idea that gap junctions can alter neuronal excitability and firing threshold.

## 4. Discussion

Our simulations have demonstrated that gap junction blockade generally produces a decrease in neuronal synchrony when applied exclusively to the nucleus of interest and either an increase or decrease in neuronal synchrony when decreases in gap junction conductance and input current are combined. Traditionally, gap junctions have been proposed to be a mechanism for synchronizing neuronal activities [33]; however, experimental studies have demonstrated that blockade of gap junctions may either decrease or increase neuronal synchrony [24, 25]. 

A prior study using HH-style neurons also investigated neural synchrony via gap junctions [1]. While the focus of this study was on the intrinsic properties of neurons and not the modification of gap junction current itself, a potential inhibitory role for gap junctions on synchrony was proposed based on interactions with strong *I*_*Na*,*p*_ currents. In our model, and HMs in general, *I*_*Na*,*p*_ is more modest; thus, we did not observe a similar effect. Furthermore, since the focus of our study was on changes in gap junction conductance and upstream synchrony, both of which were not included in the study by Pfeuty et al. [1], our proposed explanation for the findings of Bou-Flores and Berger [24] does not appear to overlap with the mechanism identified by Pfeuty et al. [1] and therefore can be considered an alternative explanation for an increase in synchrony with gap junction blockade in coupled neurons without high *I*_*Na*,*p*_.

While mixed results have been reported in the literature, the mechanisms underlying these differences were not identified. Thus, our simulations provide new mechanistic insight explaining these differences.

It should be noted, however, that at the onset of this study, we did not expect neuronal synchrony to increase with simulated gap junction blockade. While our observations verify that an increase in synchrony can occur with gap junction blockade, it certainly defies intuition. Previous computational models of oscillatory networks have shown that while strong gap junction coupling can synchronize neuronal oscillations, weak gap junction coupling can phase-lock cells [34, 35], the later of which could potentially lead to an increase in neuronal synchrony. An alternate explanation, however, must be considered when taking into account the methods employed for application of the gap junction blockers in the experimental studies described above. In this case, we reasoned that since the gap junction blockers were applied directly to the artificial cerebrospinal fluid bathing the tissues, they might have affected CNS areas other than the motoneuron nucleus responsible for the motor output studied. If this were the case, the synaptic input to the hypoglossal and/or phrenic motoneuron nuclei would potentially be reduced. Assessment of this possibility revealed that simultaneously reducing the gap junction conductance and the input current that corresponds to the input from the upstream drivers can produce in an increase in neuronal synchrony, an effect that was distinctly different from that observed when reducing only gap junction conductance of the motoneuron nucleus. Thus, our computational model and simulations clearly demonstrate that gap junction blockade can decrease or increase neural synchrony depending on the circumstances associated with drug application.

Our simulations also demonstrated that firing frequency remained constant during simulated gap junction blockade. It should be noted, however, that some neurons occasionally failed to fire under high gap junction conductances. This behavior was not observed in the absence of gap junction coupling; therefore, we speculate that coupling neurons with sufficiently high conductance may not only contribute to synchrony, but sufficiently out of phase neurons may also inhibit one another. In addition, when the input current was set near the firing threshold (i.e., rheobase) of single neurons, excitability was dramatically reduced. In this case, it should be pointed out that significant gap junction coupling can effectively reduce excitability by creating a “super cell,” where neurons are so highly coupled that they function as a singular cellular entity. While this would increase the firing threshold of neurons in the network, it would not necessarily alter other electrophysiological properties of the neuron once firing. Thus, changes in excitability from gap junctions may not necessarily translate into changes in firing frequency.

Gap junction blockers may also exert pharmacological effects independent of their gap junction-mediated effects that lead to alterations in neuronal excitability. Thus, under experimental conditions, a decrease in input current could be produced by nonspecific effects of gap junction blockers [36, 37]. Furthermore, while local blockade of gap junctions decreases the synchrony of the local neuronal network, distant blockade of gap junctions or nonjunction actions of the uncoupling agents used could mask the local gap junction-dependent effects on synchrony through a modification of the input current to the neurons or nucleus of interest. As parallel to our neuronal networks, we can consider the case of the AV and SA nodes of the heart, where pacemaker cells are weakly coupled to themselves and surrounding tissue, but increased coupling can create enormous load, decreasing excitability. Decreased coupling of these cells provides less excitation to downstream cardiac regions, also leading to conduction failure [38, 39]. A similar interpretation can potentially be applied in future studies of neuronal gap junction coupling.

While for our model, we have presumed excitatory synaptic projections, the biological situation for many rhythmically driven nuclei may also include inhibitory projections. In this case, blockade of gap junctions could contribute to alternate or additional behaviors not seen in the current study when synchrony is perturbed. Further, blockade of gap junctions in the biological situation would also affect glia, which are known to contain extensive gap junction coupling [6], and the uncoupling of glia could contribute to behaviors in the system that we were not capable of capturing with the present model. It would be of considerable general interest to model the extentto which such coupling strength between an excitable neuron and a nonexcitable glial cell might affectneuronal network activity [40], and future studies should consider this possibility.

 We suggest, however, that whether synchrony is reduced or enhanced potentially relies more heavily on the nature of the incoming inputs into a nucleus rather than from a reduction of synchrony in the nucleus itself. 

In the current study, we have also kept our model fairly general. We have not included intracellular processes that could potentially contribute to a variety of effects that might appear in real neurons. Additionally, we did not address alterations to ion channel conductances in our simulations although different concentrations of ion channels known to play roles in firing frequency and bursting behavior, such as *I*_*SK*_,  *I*_*Na*,*p*_ and voltage-gated calcium currents [1], may exert an unforeseen effect on synchrony. Our model of gap junctions was also kept deliberately simple in order to avoid potentially more exotic effects that might be seen in more elaborate models [41]. While this may limit our ability to speak in terms of specific Cx proteins and their contribution to synchrony, we are satisfied with the capacity for even an incredibly simplistic gap junction model to potentially lend insight into a number of seemingly conflicting observations from biological experiments.

Though gap junctions have traditionally been viewed as simple synchrony enhancers, and some of our simulations appear to support this view, our study has highlighted the idea that the role of gap junctions can be deeply nuanced and highly dependent on the state of the cell and surrounding tissue. As is common in biology, conflicting experimental results from different preparations and/or laboratories may not be an indication of a faulty experimental paradigm so much as a nascent understanding of all processes underlying a behavior. It is our desire that with this new information, experimental biologists might renew their interest in investigating the curious effects of gap junction blockade and further investigate the consequences of upregulating and/or downregulating these unique neural coupling proteins.

## Figures and Tables

**Table 1:** Firing frequency statistics.

Firing frequency	Mean	Standard deviation
Before	21.0860	0.4608
During	21.4072	0.0361
Late	21.2578	0.1467

**Table 2:** Firing frequency statistics.

Firing frequency	Mean	Standard deviation
Before	13.7671	0.0226
After	12.8132	0.0684
